# Association of CMV genomic mutations with symptomatic infection and hearing loss in congenital CMV infection

**DOI:** 10.1186/s12879-019-4681-0

**Published:** 2019-12-10

**Authors:** G. Clement Dobbins, Amit Patki, Dongquan Chen, Hemant K. Tiwari, Curtis Hendrickson, William J. Britt, Karen Fowler, Jake Y. Chen, Suresh B. Boppana, Shannon A. Ross

**Affiliations:** 1 Department of Pediatrics, The University of Alabama School of Medicine, CHB 116, 1600 6th Avenue South, Birmingham, AL USA; 2 Department of Biostatistics, The University of Alabama School of Public Health, Birmingham, AL USA; 3 Informatics Institute, The University of Alabama at Birmingham, Birmingham, AL USA; 4 Department of Medicine, The University of Alabama at Birmingham, Birmingham, AL USA; 5 Department of Microbiology, The University of Alabama at Birmingham, Birmingham, AL USA

**Keywords:** Cytomegalovirus (CMV), Sensory neural hearing loss (SNHL), Next generation sequencing (NGS), Viral diversity

## Abstract

**Background:**

Congenital cytomegalovirus (cCMV) infection is the most common congenital infection and a leading cause of long-term neurological and sensory sequelae, the most common being sensorineural hearing loss (SNHL). Despite extensive research, clinical or laboratory markers to identify CMV infected children with increased risk for disease have not been identified. This study utilizes viral whole-genome next generation-sequencing (NGS) of specimens from congenitally infected infants to explore viral diversity and specific viral variants that may be associated with symptomatic infection and SNHL.

**Methods:**

CMV DNA from urine specimens of 30 infants (17 asymptomatic, 13 symptomatic) was target enriched and next generation sequenced resulting in 93% coverage of the CMV genome allowing analysis of viral diversity.

**Results:**

Variant frequency distribution was compared between children with symptomatic and asymptomatic cCMV and those with (*n* = 13) and without (*n* = 17) hearing loss. The CMV genes *UL48A, UL88*, *US19* and *US22* were found to have an increase in nucleotide diversity in symptomatic children; while *UL57, UL20, UL104, US14, UL115*, and *UL35* had an increase in diversity in children with hearing loss. An analysis of single variant differences between symptomatic and asymptomatic children found *UL55* to have the highest number, while the most variants associated with SNHL were in the *RL11* gene family. In asymptomatic infants with SNHL, mutations were observed more frequently in *UL33* and *UL20*.

**Conclusion:**

CMV genomes from infected newborns can be mapped to 93% of the genome at a depth allowing accurate and reproducible analysis of polymorphisms for variant and gene discovery that may be linked to symptomatic and hearing loss outcomes.

## Background

Cytomegalovirus (CMV) is a ubiquitous infectious agent resulting in significant morbidity in immunocompromised hosts and is also the most frequent cause of congenital infection. Congenital CMV infection (cCMV) is a leading cause of sensorineural hearing loss (SNHL) and neurodevelopmental disabilities. Of the approximately 30,000 infants born in the U.S. each year with cCMV, 10–15% (3000 to 4500) develop permanent neurological and sensory sequelae, with SNHL being the most common [1].

CMV is one of the largest human virus pathogens with a genome of 235 kb and over 200 open reading frames [2–4]. Studies that examined a limited number of genes including those coding for envelope glycoproteins gN and gB demonstrated extensive genetic variability among virus isolates and showed that infected individuals including infants with cCMV harbor multiple virus strains [5–7]. Recent studies utilizing next-generation sequencing (NGS) technology have provided evidence for inter- and intra-host genetic diversity among infected individuals, establishing that genome-wide mutation and recombination rate maps for CMV to be more divergent than other herpesviruses [4, 8–11]. This study utilizes whole viral genome NGS of specimens from congenitally infected infants to explore if viral variants are associated with symptomatic infection and SNHL.

## Methods

### Patient population

Samples were obtained from infants who participated in a multicenter natural history study of cCMV (CHIMES Study) [12, 13]. Newborns were screened for cCMV within the first few days of life by testing saliva specimens; and CMV was confirmed in infants with positive screening results on the basis of a positive urine and/or saliva sample obtained within the first 3–6 weeks of life by rapid culture or PCR [12, 13]. A convenience sample of 30 study subjects from all seven study sites were selected based on the availability of adequate remnant urine specimens (at least 200 ul) obtained within the first 3–6 weeks of life (median age of urine collection 21.5 days, range 2–114 days). There were no differences in the demographic characteristics between the study infants and the entire CHIMES cohort. Infants were considered to have symptomatic cCMV if they had any of the following findings in the newborn period: generalized petechial rash, purpuric rash, hepatomegaly, splenomegaly, and jaundice with direct bilirubin of 3 mg/dL or greater, unexplained neurologic/CNS abnormalities (e.g., microcephaly, seizures, focal or generalized neurologic deficits), or chorioretinitis. More symptomatic infants were sampled for this study to enrich for the hearing loss outcome. However, the frequency and severity of symptoms did not differ between the study cohort and the entire CHIMES cohort. A non-sedated auditory brainstem response (ABR) was obtained at the initial enrollment visit at 3–8 weeks of life, and the study children were then monitored every 6 months during the first 4 years of life for hearing loss using age-appropriate audiological testing protocols [14]. Among the 30 study infants, 13 had symptomatic infection and 17 were asymptomatic. Two children had normal hearing at the enrollment visit for ABR testing and were lost to follow-up. The remaining 28 children completed audiological testing at a median age of the last audiological test at 48 months (range 7–48 months). Seven of the 13 symptomatic infants and 6 of 17 asymptomatic infants had SNHL. Six study children received antiviral therapy; however, samples for sequencing were collected before the start of antiviral therapy.

The study was approved by the University of Alabama at Birmingham Institutional Review Board for Human Use (IRB) (Approval number: X120625004). Written informed consent was obtained from a parent for their newborn’s enrollment in the study.

### Samples and DNA purification

Urine specimens obtained from infants at one time point, the enrollment visit, were used for NGS. Samples were centrifuged at 1200 rpm, 4 °C, for 5 min; and DNA was extracted from the supernatant using the Qiagen Viral RNA kit. As downstream applications require 100 ng of DNA and evidence suggests that low quality or lower amounts of DNA may not give reliable results, all samples were whole genome amplified (WGA) (Repli-G Qiagen) and quantitated by Qubit dsDNA BR Assay. Studies have demonstrated that sequence coverage and alignment error rates are in a similar range with genomic DNA using this technique [15, 16]. Samples from each infant were fragmented to 150–200 base pairs (bp) and indexed. The Agilent SureSelectXT Custom 0.5–2.9 Mb Target Enrichment library with capture probes [17–22] was designed based on the Merlin strain and 87 recently discovered clinical strains [4], and used to select the CMV DNA following the manufacturer’s instructions. NGS was performed using the 2 × 150 cycle paired-end sequencing protocol on the Illumina MiSeq platform [23] .

### Data analysis

Paired reads were imported from Illumina basespace and those that did not meet Illumina quality control filter were removed. Adaptors were trimmed and duplicate reads were removed. The remaining reads were assessed for quality: the sequence files containing the quality scores from the base-caller algorithm was employed for further trimming in which the modified Mott trimming algorithm was employed: quality scores (based on a Phred quality score defined as: Q = −10Xlog10(P), where P is the base-calling error probability) were used to calculate the limit for which bases to be trimmed. Here, Q is set to an error probability where p_error_ = Q ^-Q/10^. For every base a new value is calculated equal to limit – p_error_, such that any value less than zero is removed since the negative values will be of low quality with high error probability. In this study the limit scores were set to ≤ 0.5; hence, any base with a -p_error_ value greater than this limit within the reads were trimmed (CLC Genomics Workbench 10 (Qiagen)) [21, 24–28].

To aid in the phylogenetic analysis of key genes, an iterative mapping technique was employed to assemble sequences as previously described [4, 29]. Both the hierarchical likelihood ratio test (which analyzes the goodness-of-fit between models using the neighbor joining construction method to account for a varying rate of evolution, with a confidence level of 0.01) and the Bayesian information criterion (a rank substitution model) were tested on five models: Jukes-Cantor, Felsenstein 81, Kimura 80, Hasegawa-Kishino-Yano, and General Time Reversible. Both models indicated the Kimura 80 was optimal based on the p and BIC values respectively. Hence, phylogenetic tree analyses were performed using the Kimura 80 model with 1000 bootstrap replicates [30] (CLC Genomics Workbench 10 (Qiagen)).

To compare variants across cohorts, the reference strain Merlin was used for mapping: match score of 1; a mismatch cost of 2 (irrespective of the type of substitution); an insertion cost of 3; a deletion cost of 3 with a length fraction of 0.5 and a similarity fraction of 0.8. The CLC genomics workbench low frequency variant detector that models both the samples and the sequencing errors was employed to analyze a minimum coverage of 15 unbroken reads (indicating that 15 quality control reads must be present at the nucleotide position to call a variant), a count of at least 2 reads per variant (meaning at least two reads must show that variant to be called), with a 1% relative read direction filter [31–33]. Variants were only considered for analysis if they were detected at a threshold cutoff frequency of ≥1%; these validated thresholds reduce carryover of errors by base-calling and amplification and indicates less than 1% chance of an error occurring [34–36].

To verify these parameters for downstream analysis, two internal controls were run. The first was a limited passaged BAC clone for the CMV strain TR, which had an error rate of 0.03%, between two runs. The error rate was calculated by running the sample through the pipeline from the beginning and comparing the variants found at any location between the two runs divided by the number of possible locations, reported as a percentage [37–39]. Second, to confirm the reproducible nature of the pipeline from DNA isolation to variant calling, a patient sample was prepared and sequenced three times from the beginning of the pipeline resulting in an error rate of 0.09% of variants called in the genome between runs [8, 20, 29]. To determine whether a variant was synonymous or non-synonymous, CDS annotations of the Merlin strain were used in coordination with the variant caller. If a single nucleotide variant (SNV) occurred in the same position as a multi-nucleotide variant (MNV), the variants were reported separately, as the variants may be found in other case samples (CLC Genomics Workbench 10 (Qiagen)). The nomenclature is from http://www.hgvs.org/mutnomen/.

For analyzing patients across multiple groups (symptomatic, asymptomatic, normal hearing, SNHL and asymptomatic with SNHL), each sample genome was inspected to determine if at least 15 quality-controlled reads existed for all variants compared between samples. To compare only the highest quality variants between patient samples, any variant with a quality score less than 20 was removed.

Under this conservative criterion, 7% of the genome failed to match adequately with the reference genome consistently over all 30 samples. These regions of high variability (Additional file 2: Table S1), were similar to those previously reported [4, 8–11, 20, 40] and were removed from comparisons between samples. The remaining 93% of the genome had consistent depth of coverage and variant calling, allowing for the subsequent analysis between cohorts.

Following the phylogenetic analysis of well studied genes in CMV (*UL55* and *UL73*), the diversity of the virus was further evaluated between the groups by analyzing the distribution of variants in each cohort (variant density). Custom scripts were generated to measure nucleotide diversity (π) [41] and mean diversity [42] for each gene and compared between the groups using the Mann-Whitney Wilcoxon Test [43]. To further evaluate novel regions that may be linked to sequelae, a variant and principal component analysis was used to identify relationships among the mutation density, genes affected, and patient symptoms. Raw sequence files were used to align to merlin genome (AY446894) after removing adapter sequences, duplicates were marked, sorted and indexed as above. To carry out this analysis a similar low frequency variant detection using Partek Genomic Suite (PGS, Partek, LLC, MO, USA) was employed. The SNP density was determined 2 k bps ahead of transcription start site and inside the gene. A Fisher exact test was used to correlate clinical outcomes. A gene list with significant association of specific variants with symptomatic infection, hearing loss and major trends within the data set were determined with measurements of relative inertia ordered to their position along the different axes and correlated between the axes and the original variables. This list was then clustered to generate a heatmap and principal component analysis (PCA) plots.

To measure differences in the frequency distribution of individual variants and variant density between the groups of infants, a standard case-control analysis using Fisher’s exact test was performed [44–49]. To facilitate the interpretation of non-synonymous variants for which structures of proteins have been resolved and published, files from the Protein Data Bank (PDB), http://www.rcsb.org/ were downloaded and analyzed.

## Results

### Next-generation sequencing of CMV complete clinical genomes

As characterizing CMV genome variability from NGS data is challenging, a multi-prong strategy was implemented in which overall variation was analyzed followed by phylogenetic analysis of previously well-studied genes. Analysis of variability, depth of coverage and variant calling indicated 93% of the genome was consistent to accurately compare variants between symptomatic and asymptomatic children as well as those with SNHL and normal hearing. Table 1 lists general sequencing and variant calling results. CMV variability along the genome was similar in infants with symptomatic cCMV, asymptomatic cCMV and children with hearing loss. The regions with the most variation in all three groups were between unique long *(UL)1* and *UL11, UL33, UL37, UL55, UL73, UL74, UL119* and *UL120*, as well as in areas preceding the internal repeat short (IRS) and terminal repeat short (TRS) domains (Additional file 1: Figure S1). Genes encoding glycoproteins had 8892 variants; genes encoding viral genome replication and regulation of viral gene expression contained 4697 variants; and genes involved in immune regulation had 4144 variants. The regions with fewer variants were found in genes encoding capsid proteins (1196 variants). The coding regions with the least variation were seen in *UL46, UL85* and *UL103*.

**Table 1:** Summary of next generation sequencing data from urine of infants with congenital CMV

No. of patient samples	30
No of bases sequenced	7.4 × 10^9^
Average reads mapped to reference per sample	2.1 × 10^6^
Average maximum depth	44,063
Average depth	1468
Average genome coverage	93%
Total variants	172,171
Unique variants	42,846
Single nucleotide variants (SNV)	29,306
Multi-nucleotide variants (MNV)	2517
Insertions	1905
Deletions	1204
Nucleotide replacements	174
Urine Viral Load Median (range)	16,424 (69–6.4 × 10^5^)IU/mL

### CMV diversity associated with symptomatic infection

In the initial analysis all SNVs, multi-nucleotide variants, insertions-deletions and nucleotide replacements were compared between children with symptomatic and asymptomatic cCMV (Additional file 1: Figure S1). The average number of variants was similar between symptomatic (5485 per sample) and asymptomatic infants (5981 per sample). This analysis indicated that 7% of the genome failed to consistently map at a depth reliable to call variants among all 30 samples and were excluded from further analysis (Additional file 2: Table S1).

**Fig. 1:**
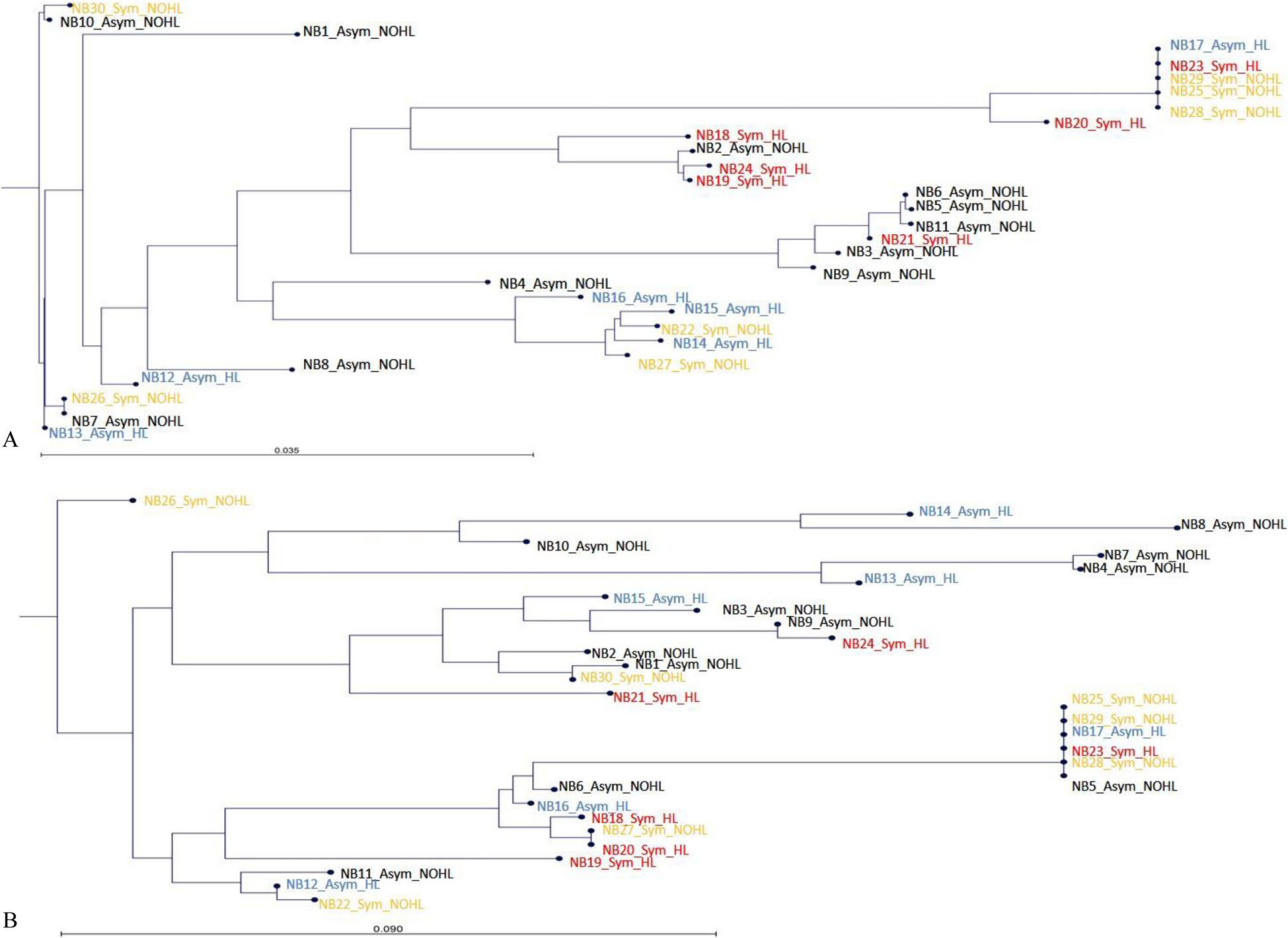
Phylogenetic analysis of CMV envelope glycoproteins gB and gN consensus sequences. **a** A maximum phylogenetic tree was constructed from consensus sequences generated for *UL55* from each patient. **b** Similar analysis was carried out for *UL73* (asymptomatic newborns with normal hearing in black; asymptomatic newborns with hearing loss in blue; symptomatic newborns with normal hearing in orange; and symptomatic newborns with hearing loss in red). Abbreviations: NB, new born; Sym, symptomatic; Asym, asymptomatic; NOHL, normal hearing; HL, hearing loss

As envelope glycoproteins gB (*UL55*) and gN (*UL73*) have been extensively studied in relation to cCMV disease outcome [50–55], maximum-likelihood phylogenetic trees of these genes were constructed from extracted consensus sequences from all samples (CMV_UL73_align.tar.gz; CMV_UL55_align.tar.gz). There was no correlation to symptomatic outcome as the consensus sequences for the samples were interspersed among the trees in both genes (Fig. 1a and b).

To further analyze genetic diversity, nucleotide diversity was calculated for each gene [41] and compared between symptomatic and asymptomatic children using a non-parametric analysis. The greatest difference was seen in *US8*, a cytoplasmic glycoprotein, with the higher diversity in the asymptomatic group. No association between symptomatic infection and variants within specific genes was observed.

As CMV transcription is a complex process with alternative start sites, a principal component analysis (PCA) was employed to find similarity based on variances of the SNP density in all samples for each coding region including 2 k bps ahead of the transcription start sites. For novel gene discovery, the SNP density was determined 2 k bps ahead of transcription start sites. The Principle Component Analysis was used to show similarity based on variances of all values (SNP density) of all samples. Each principle component (PC1, 2, 3…) with PC1 and PC2 indicating the most variance were included to show similarity/dissimilarity of samples from highest to the lowest.

**Fig. 2:**
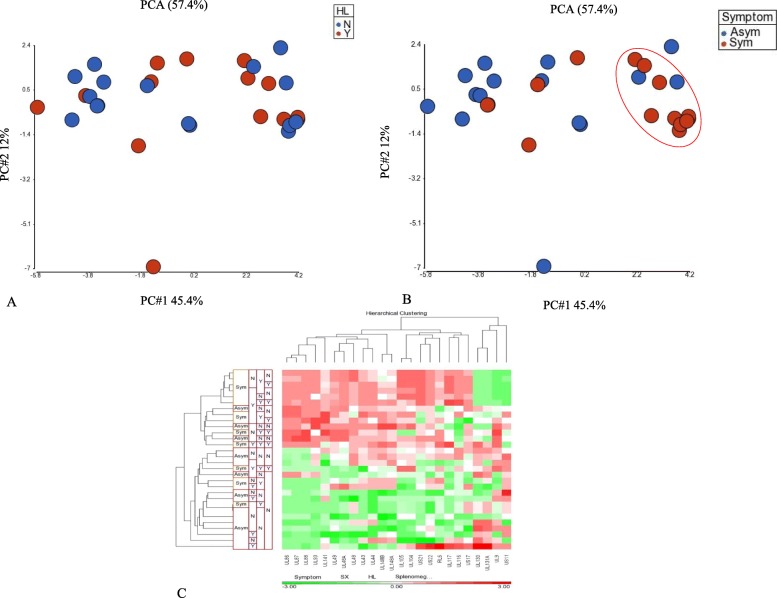
Principal component analysis for patterns among clusters of variants and symptoms. For novel gene discovery, the SNP density was determined 2 k bps ahead of transcription start sites. The Principle Component Analysis (PCA) was used to show similarity based on variances of all values (SNP density) of all samples. Each principle component (PC1, 2, 3…) with PC1 and PC2 indicating the most variance were included to show similarity/dissimilarity of samples from highest to lowest. A Fisher exact test was used to correlate clinical outcomes and generate a significant gene list clustered with hearing loss (HL) (**a**) or symptomatic infection (**b**). **c** A heatmap displaying hierarchical clustering performed on samples (horizontal axes) and genes (vertical) with high level SNP densities associated with outcomes. The color indicates SNP density values above (red) average and below (green) average (no color) after z-normalization (average = 0 and SD = 1)

A Fisher’s exact test was then used to determine the association with clinical outcomes, generating a gene list that showed significant association between specific variants and symptomatic infection and major trends within the data set. A heatmap displaying hierarchical clustering performed on the samples and genes was used to show the SNP density levels associated with clinical outcomes. Of the possible clinical outcomes, the only group with *p*-values less than 0.05 were seen between the symptomatic and asymptomatic infection and not between SNHL and normal hearing (grouping in Fig. 2a and b). Variants associated with *UL48A* had the lowest p-value associated with symptomatic infection (Fig. 2c and Additional file 3: Table S2).

**Fig. 3:**
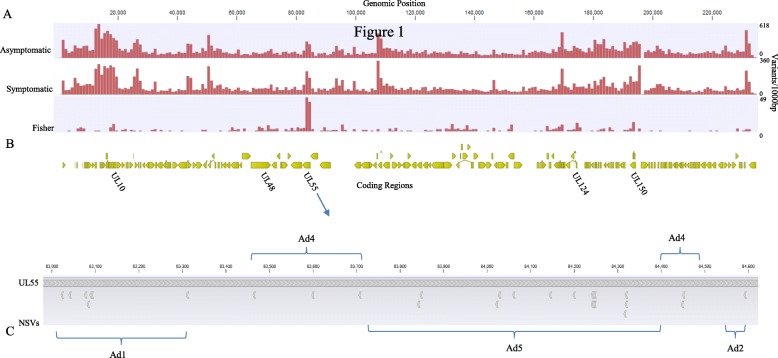
CMV variants in symptomatic and asymptomatic children. **a** Variant density of CMV genomes isolated from newborns (17 asymptomatic, 13 symptomatic) calculated in 1000 bp windows with Merlin as the reference strain. **b** Variants more frequent in symptomatic infection (Fisher’s exact test *p* < 0.05) are plotted with the genome position. The coding regions with the highest number of such variants are listed in their relative genomic position. **c** UL55 NSVs in relation to known antigenic domains (AD). Top panel shows the amino acid sequence of CMV strain Merlin. Bottom panel shows where NSVs (indicated by arrows below the reference strain) are more likely seen in infants with symptomatic congenital CMV in relation to antigenic domains

To identify specific variants more common in infants with symptomatic infection, a case-control analysis using Fisher’s exact tests was performed. There were 502 variants significantly more frequent in symptomatic infants (*p* < 0.05) (Fig. 3). Among these, 419 were in 76 known coding regions: 125 variants were found in glycoprotein genes; 84 in genes encoding proteins responsible for viral DNA packaging, replication and regulation; 75 in coding regions involved in viral capsid formation; and 27 were in genes involved in CMV morphogenesis. Of the 419 variants in these coding regions, 132 resulted in NSVs. A complete list of the variants in symptomatic infection is shown in Additional file 4: Table S3.

*UL55*, encoding the envelope glycoprotein gB, was found to have the highest number of genomic variants (*n* = 92) in symptomatic cCMV, with 33 of the variants resulting in NSVs. Analysis of the *UL55* protein structure indicates all NSVs were in known antigenic domains or the signal sequence domain (Fig. 3c).

Other genes with specific variants more frequent in symptomatic cCMV include 34 variants in *UL48* encoding a tegument protein involved in virus entry, spread, regulation of immune signaling pathways and virion assembly [56]. Twenty one variants were observed in *UL10,* encoding a highly glycosylated transmembrane immunomodulatory protein, and 16 in *UL150*, a diverse region with unknown functions [4, 9].

### CMV variants associated with SNHL

Overall CMV genomic diversity was similar in the group of 13 children with SNHL (5782 variants per sample) and 17 with normal hearing (5706 variants per sample). Phylogenetic analysis revealed that hearing loss outcomes were interspersed throughout the trees in both *UL55* and *UL73* (Fig. 1a and b). A comparison of CMV nucleotide diversity by gene in samples between infants with normal hearing and those with SNHL revealed a significantly higher diversity (*p* < 0.0007) in 6 genes: *UL57, UL20, UL104, US14, UL115,* and *UL35* in children with hearing loss (Additional file 5: Table S4).

**Fig. 4:**
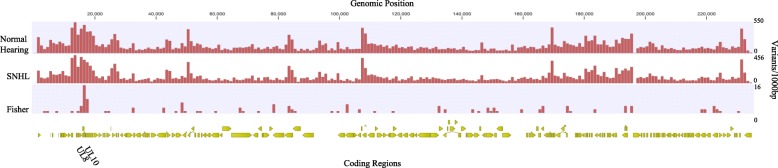
CMV variation from children with normal hearing and SNHL. Variant density of CMV genomes isolated from 17 children with normal hearing and 13 children with SNHL calculated in a 1000 bp sliding window with Merlin as the reference strain. Variants more frequent in viruses from children with SNHL (Fisher’s exact test *p* < 0.05) are plotted with the genome position. The coding regions with the highest number of variants are listed with the genomic position

A comparison of specific variants indicated 131 polymorphisms in 38 coding regions were more frequent in those with hearing loss (*p* < 0.05), 35 resulting in NSVs. The most variants and NSVs in samples from children with SNHL were found in the *UL8* coding region and other members of the *RL11* gene family (Fig. 4 and Additional file 6: Table S5).

**Fig. 5:**
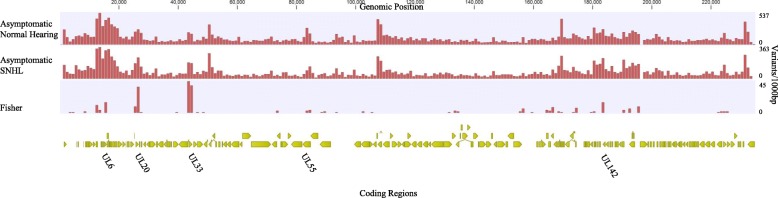
CMV variants from asymptomatic children with normal hearing and SNHL. Variant density of CMV genomes isolated from 11 asymptomatic newborns with normal hearing and 6 asymptomatic newborns that developed SNHL calculated in a 1000 bp sliding window with Merlin as the reference strain. Regions with variants more frequent in viruses from children with asymptomatic infection and SNHL (Fisher’s exact test *p* < 0.05) are plotted with the genome position

Among children with asymptomatic cCMV, a comparison of the variants between the group with SNHL and those with normal hearing revealed 255 variants in 36 coding regions including 92 NSVs that were more frequent in children with SNHL. The highest number of variants were in *UL33* (*n* = 84), encoding a seven-transmembrane spanning putative chemokine receptor. *UL20*, encoding a polymorphic glycoprotein believed to sequester cellular proteins for lysosome degradation, had 28 NSVs in asymptomatic children with SNHL [4, 57]. Multiple variants were also seen in *UL55* (Fig. 5 and Additional file 7: Table S6).

## Discussion

Cytomegalovirus is the most common cause of congenital newborn infection in the United States [58]. The pathogenesis of CMV-related SNHL and neurodevelopmental disabilities is poorly understood; and in asymptomatic cCMV, there are no surrogate markers to identify children at risk for hearing loss. In this study, CMV genomes from urine samples of infants with cCMV were sequenced by NGS to identify viral diversity and determine if viral diversity is related to newborn disease and SNHL. This study utilized a bait library based on 88 CMV strains, an extensive CMV probe design via a proven commercial target enrichment strategy [18, 20, 21]. This approach resulted in accurate and reproducible sequencing of CMV genomes from clinical specimens mapping 93% of the genome at a depth to compare diversity between the groups of infants with and without newborn disease and hearing loss.

As in previous studies [4, 9, 11], areas of high diversity were observed between *UL1* and *UL11*, and near *UL33, UL37, UL55, UL73, UL74, UL119*, and *UL120* as well as in the genome preceding the IRS and TRS domains. Although the diversity of genes encoding envelope glycoproteins *UL55, UL73* and *UL74* has been established by traditional sequencing methods, deep sequencing analysis by Sijmons et al. [4, 29] and Renzette et al. [8, 9, 11] have demonstrated areas of high diversity in other regions of the CMV genome. The consistency of these findings among various studies and populations highlights the need for studies to define the importance of these highly variant regions in the pathogenesis of cCMV.

The overall frequency of CMV genomic variation was similar among infants with symptomatic and asymptomatic cCMV and those with and without hearing loss. Previous work based on CMV population genetic modeling from congenitally infected infants and transplant patients have demonstrated that viral populations may range from stable to highly divergent over time and among tissue compartments [3, 4, 9–11, 20, 40]. Similarly, a recent study by Hage et al. found evidence of genome switching in half of transplant recipients with no correlation with fatal outcomes detected [20]. These CMV population studies have also shown a wide variation in nucleotide diversity ranging from 0.021 to 0.25 [4, 8–11]. In this study, phylogenetic analysis of *UL55* and *UL73* consensus sequences demonstrated that symptomatic infection and hearing loss outcomes were interspersed, with no unique consensus sequence(s) in infants with symptomatic infection or SNHL. Previous studies examining CMV glycoproteins and other loci as prognostic markers for SNHL have also reported conflicting results with no consistent linkage between specific strains and sequela [50, 55, 59].

Although results from this study found similar areas of high diversity as other groups [4, 8–11, 40], 93% of the genome could be compared among samples with high confidence. Following the phylogenetic analysis of the two well studied genes gB and gN, the association of the overall gene variation with clinical outcomes was examined with PCA analysis and the Mann-Whitney Wilcoxon Test. Although there was no significant association between nucleotide diversity and specific gene variation, PCA analysis of the coding regions including the 2 kb region ahead of the transcription start site found several genes with increased SNP density associated with symptomatic outcome (Fig. 2 and Additional file 3: Table S2).

To further evaluate potential individual variants that may be linked to symptomatic outcome and hearing loss, a second Fisher’s exact test was employed to examine if specific variants were associated with symptomatic infection and SNHL. The largest number of specific variants and NSVs in any single coding region associated with symptomatic infection were in the gene encoding the extensively studied envelope glycoprotein gB (*UL55*). All of the NSVs found in gB were in the signal sequence and known antigenic domains [60], near or within sites where mutations have been shown to reduce binding of both neutralizing and non-neutralizing antibodies (Fig. 3c) [61–63]. Previous studies have suggested that increased CMV diversity could lead to the generation of CMV variants with improved ability to replicate in distinct host compartments [11, 40, 64, 65]. As gB is critical for attachment, entry, and cell-to-cell spread of the virus, variation in gB may enhance the ability of the virus to replicate and spread by altering antibody binding leading to more severe disease. Although antibody binding sites in gB have been studied, questions remain as to whether mutating these sites alters protein conformation, or if the sites would be similar when gB was in the prefusion or postfusion conformation [60–63, 66]. Future mutagenesis studies of the antigenic domains may consider the NSVs described here in antibody mapping of the altered conformational states of gB, as well as in studies exploring cCMV pathogenesis. Furthermore, a larger sample size of infants with cCMV as well as functional studies are needed to determine whether these variations could serve as markers of outcome. These findings may also inform vaccine design by identifying regions that are important for virus fitness.

Although SNHL is the most common sequelae of cCMV second only to genetic causes in childhood deafness, the pathogenesis of SNHL in children with cCMV is not understood. As only a minority of asymptomatic children develop hearing deficits, clinical and/or laboratory marker(s) for early identification of children at the highest risk of hearing loss are needed for early identification, targeted monitoring and timely intervention. Although phylogenetic analysis did not indicate any consensus sequence linked to SNHL, the Mann Whitney Wilcoxon test found several genes with increased nucleotide diversity correlated to SNHL (Additional file 5: Table S4). The gene with the highest nucleotide diversity associated with SNHL was *UL57*, involved in DNA replication and known to be a common target in antibody responses [67]. The most variants and NSVs in samples from children with SNHL were found in the *UL8* coding region and other members of the *RL11* gene family (Fig. 4 and Additional file 6: Table S5). The UL8 protein has recently been found to be a conserved Ig-like domain, that interacts with a surface molecule present on activated neutrophils, reducing proinflammatory cytokines potentially acting as an immunosuppressive [68].

Among asymptomatic children who develop SNHL, the most NSVs were found in *UL20* and *UL33. UL20* encodes a polymorphic glycoprotein hypothesized to degrade several cellular components aiding immune evasion [4, 57]. The gene has also been found to be highly divergent in other NGS studies [4], and the role of the protein in immune evasion may point to a potential cause for delayed SNHL. *UL33* encodes a seven transmembrane spanning protein and putative chemokine receptor [69]. Much more work is needed to determine whether these genes and specific CMV polymorphisms found here may play a role in pathogenesis or potentially serve as markers for sequelae.

A limitation of this study is that only a small proportion of the infected children identified in the CHIMES study were included, which may have led to selection bias. However, this potential selection bias is unlikely because the demographic characteristics were similar between the 30 study children and the entire CHIMES cohort. Urine samples obtained in the newborn period were analyzed in this study and children were followed for 4 years for hearing outcome. Previous sequencing work has demonstrated that viral population change over time, thus the relationship between viral mutations found in the neonatal period may not be associated with long-term adverse outcome. Future studies should evaluate how the change in viral populations/diversity over time correlate with delayed onset SNHL. Moreover, studies assessing CMV variation from maternal and infant virus and the relationship with symptoms and delayed SNHL would be informative.

In addition, approximately 7% of the genome could not be mapped accurately to the reference genome for variant calling and comparisons between the groups. Other NGS studies have also reported these areas to be highly diverse and difficult to map as expression of these genes is complex with alternate transcriptional start sites overlapping with other annotated genes [4, 9, 10].

Nonetheless, the strategy employed in this study shows that 93% of the genome from clinical samples can be successfully mapped at a depth allowing accurate and reproducible analysis as well as comparisons of polymorphisms from CMV infected patients. This coverage is consistent with other studies in which approximately 89.5–97% of the viral genome was successfully sequenced [4, 8, 11, 70]. More recent reports suggest that these regions of high diversity that fail to map consistently are most likely from recombination events in multiple strain infected individuals [17, 22]. However, by analyzing the more stable regions of the CMV genome from infected newborns with well-defined outcomes, a number of genes and specific variants were found that will be a focus in future studies to ascertain if these mutations and residues in proximity are linked to cCMV disease and SNHL.

As the first study to explore viral variations at a genome-wide level associated with symptomatic cCMV and hearing loss, these findings will need to be confirmed with future NGS analyses on larger sample sizes as well as with improved algorithms for comparing diverse variant strains and the diverse regions within those strains. Furthermore, functional studies will need to be conducted to define whether CMV diversity and specific polymorphisms play a role in disease pathogenesis, including the use of animal models to assess the relationship between genetic variants and CMV pathology. Thus, the findings in this study represent a step in understanding the impact of CMV genetic variation on disease and in the identification of infants with cCMV at increased risk for adverse outcomes.

## Conclusions

CMV genomes from infected newborns with well defined outcomes can be isolated from urine samples and mapped to 93% of the genome at a depth allowing accurate and reproducible analysis of polymorphisms for variant and gene discovery. This analysis found genes and specific variants that are associated with symptomatic and hearing loss outcomes representing a further step in understanding how CMV genetic variation may impact disease as well as identifying potential markers in infants with cCMV at increased risk for adverse outcomes.

## Supplementary information

**Additional file 1: Figure S1.** All unique CMV variants in the 30 newborns (top panel) along with those divided into each group. The number of unique variants are listed in each group on the left with the maximum number of variants in any 1000 base pair window on the right. Bottom panel is schematic representation of CMV coding regions based on the reference strain Merlin along with regions with the most variation.

**Additional file 2: Table S1.** Regions excluded from Fisher’s exact test analysis. A number of samples lacked 15 quality reads in the indicated regions, and therefore were excluded in calculating the frequency distribution of variants from all samples.

**Additional file 3: Table S2.** Table of gene regions correlated with symptoms based on principal component analysis for patterns among clusters of variants and symptoms.

**Additional file 4: Table S3.** Table of CMV variants more common in symptomatic children. The accession number prefix is listed; and nomenclature used for reporting is taken from http://www.hgvs.org/mutnomen/.

**Additional file 5: Table S4.** Non-parametric test comparing CMV nucleotide diversity by gene in samples between infants with normal hearing and those with SNHL.

**Additional file 6: Table S5.** Table of CMV variants more common in children with SNHL.

**Additional file 7: Table S6.** Table of CMV variants more common in asymptomatic children with SNHL.

## Data Availability

The data presented in this paper are available from the corresponding author on request.

## Abbreviations

AD: Antigenic domains
bp: Basepairs
cCMV: Congenital cytomegalovirus
gB: Glycoprotein B
gN: Glycoprotein N
IRS: Internal repeat short
MNV: Multi-nucleotide variant
NGS: Next generation sequencing
NSV: Non-synonymous variant
PCA: Principal component analysis
SNHL: Sensory neural hearing loss
SNV: Single nucleotide variant
TRS: Terminal repeat short
UL: Unique long
US: Unique short

## References

[1] Britt W, Remington JS, Klein JO, Wilson CB, Baker CJ (2011). Cytomegalovirus. Infectious Diseases of the Fetus and Newborn Infant.

[2] Dolan A, Cunningham C, Hector RD, Hassan-Walker AF, Lee L, Addison C, Dargan DJ, McGeoch DJ, Gatherer D, Emery VC (2004). Genetic content of wild-type human cytomegalovirus. J Gen Virol.

[3] Gorzer I, Trajanoski S, Popow-Kraupp T, Puchhammer-Stockl E (2015). Analysis of human cytomegalovirus strain populations in urine samples of newborns by ultra deep sequencing. J Clin Virol.

[4] Sijmons Steven, Thys Kim, Mbong Ngwese Mirabeau, Van Damme Ellen, Dvorak Jan, Van Loock Marnix, Li Guangdi, Tachezy Ruth, Busson Laurent, Aerssens Jeroen, Van Ranst Marc, Maes Piet (2015). High-Throughput Analysis of Human Cytomegalovirus Genome Diversity Highlights the Widespread Occurrence of Gene-Disrupting Mutations and Pervasive Recombination. Journal of Virology.

[5] Coaquette A, Bourgeois A, Dirand C, Varin A, Chen W, Herbein G (2004). Mixed cytomegalovirus glycoprotein B genotypes in immunocompromised patients. Clin Infect Dis.

[6] Pati SK, Pinninti S, Novak Z, Chowdhury N, Patro RK, Fowler K, Ross S, Boppana S, Investigators NCS (2013). Genotypic diversity and mixed infection in newborn disease and hearing loss in congenital cytomegalovirus infection. Pediatr Infect Dis J.

[7] Ross SA, Novak Z, Pati S, Patro RK, Blumenthal J, Danthuluri VR, Ahmed A, Michaels MG, Sanchez PJ, Bernstein DI (2011). Mixed infection and strain diversity in congenital cytomegalovirus infection. J Infect Dis.

[8] Renzette N, Bhattacharjee B, Jensen JD, Gibson L, Kowalik TF (2011). Extensive genome-wide variability of human cytomegalovirus in congenitally infected infants. PLoS Pathog.

[9] Renzette N, Kowalik TF, Jensen JD (2016). On the relative roles of background selection and genetic hitchhiking in shaping human cytomegalovirus genetic diversity. Mol Ecol.

[10] Renzette N, Pfeifer SP, Matuszewski S, Kowalik TF, Jensen JD. On the Analysis of Intrahost and Interhost Viral Populations: Human Cytomegalovirus as a Case Study of Pitfalls and Expectations. J Virol. 2017;91(5):9. 10.1128/JVI.01976-16 PMC5309957 27974561

[11] Renzette N, Pokalyuk C, Gibson L, Bhattacharjee B, Schleiss MR, Hamprecht K, Yamamoto AY, Mussi-Pinhata MM, Britt WJ, Jensen JD (2015). Limits and patterns of cytomegalovirus genomic diversity in humans. Proc Natl Acad Sci U S A.

[12] Boppana SB, Ross SA, Novak Z, Shimamura M, Tolan RW, Palmer AL, Ahmed A, Michaels MG, Sanchez PJ, Bernstein DI (2010). Dried blood spot real-time polymerase chain reaction assays to screen newborns for congenital cytomegalovirus infection. JAMA.

[13] Boppana SB, Ross SA, Shimamura M, Palmer AL, Ahmed A, Michaels MG, Sanchez PJ, Bernstein DI, Tolan RW, Novak Z (2011). Saliva polymerase-chain-reaction assay for cytomegalovirus screening in newborns. N Engl J Med.

[14] Fowler Karen B., McCollister Faye P., Sabo Diane L., Shoup Angela G., Owen Kris E., Woodruff Julie L., Cox Edith, Mohamed Lisa S., Choo Daniel I., Boppana Suresh B. (2017). A Targeted Approach for Congenital Cytomegalovirus Screening Within Newborn Hearing Screening. Pediatrics.

[15] Atherton RA, McComish BJ, Shepherd LD, Berry LA, Albert NW, Lockhart PJ (2010). Whole genome sequencing of enriched chloroplast DNA using the Illumina GAII platform. Plant Methods.

[16] Hou Y, Song L, Zhu P, Zhang B, Tao Y, Xu X, Li F, Wu K, Liang J, Shao D (2012). Single-cell exome sequencing and monoclonal evolution of a JAK2-negative myeloproliferative neoplasm. Cell.

[17] Cudini J, Roy S, Houldcroft CJ, Bryant JM, Depledge DP, Tutill H, Veys P, Williams R, Worth AJJ, Tamuri AU (2019). Human cytomegalovirus haplotype reconstruction reveals high diversity due to superinfection and evidence of within-host recombination. Proc Natl Acad Sci U S A.

[18] Depledge DP, Palser AL, Watson SJ, Lai IY, Gray ER, Grant P, Kanda RK, Leproust E, Kellam P, Breuer J (2011). Specific capture and whole-genome sequencing of viruses from clinical samples. PLoS One.

[19] Gnirke A, Melnikov A, Maguire J, Rogov P, LeProust EM, Brockman W, Fennell T, Giannoukos G, Fisher S, Russ C (2009). Solution hybrid selection with ultra-long oligonucleotides for massively parallel targeted sequencing. Nat Biotechnol.

[20] Hage E, Wilkie GS, Linnenweber-Held S, Dhingra A, Suarez NM, Schmidt JJ, Kay-Fedorov PC, Mischak-Weissinger E, Heim A, Schwarz A (2017). Characterization of human cytomegalovirus genome diversity in immunocompromised hosts by whole-genome sequencing directly from clinical specimens. J Infect Dis.

[21] Houldcroft CJ, Beale MA, Breuer J (2017). Clinical and biological insights from viral genome sequencing. Nat Rev Microbiol.

[22] Suarez NM, Wilkie GS, Hage E, Camiolo S, Holton M, Hughes J, Maabar M, Vattipally SB, Dhingra A, Gompels UA (2019). Human cytomegalovirus genomes sequenced directly from clinical material: variation, multiple-strain infection, recombination, and gene loss. J Infect Dis.

[23] Kozich JJ, Westcott SL, Baxter NT, Highlander SK, Schloss PD (2013). Development of a dual-index sequencing strategy and curation pipeline for analyzing amplicon sequence data on the MiSeq Illumina sequencing platform. Appl Environ Microbiol.

[24] Bowden KE, Weigand MR, Peng Y, Cassiday PK, Sammons S, Knipe K, Rowe LA, Loparev V, Sheth M, Weening K, et al. Genome Structural Diversity among 31 Bordetella pertussis Isolates from Two Recent U.S. Whooping Cough Statewide Epidemics. mSphere. 2016;1(3):15. 10.1128/mSphere.00036-16 PMC4888882 27303739

[25] Corcoran K, Sherrod CJ, Perkowski EF, Texier J, Li F, Wang IM, McVoy M, Fu TM, Dittmer DP. Genome Sequences of Diverse Human Cytomegalovirus Strains with Utility in Drug Screening and Vaccine Evaluation. Genome Announc. 2017;5(3):2. 10.1128/genomeA.01433-16 PMC5255926 28104650

[26] Lei H, Li T, Hung GC, Li B, Tsai S, Lo SC (2013). Identification and characterization of EBV genomes in spontaneously immortalized human peripheral blood B lymphocytes by NGS technology. BMC Genomics.

[27] Rasmussen AL, Okumura A, Ferris MT, Green R, Feldmann F, Kelly SM, Scott DP, Safronetz D, Haddock E, LaCasse R (2014). Host genetic diversity enables Ebola hemorrhagic fever pathogenesis and resistance. Science.

[28] Smits SL, Bodewes R, Ruiz-Gonzalez A, Baumgartner W, Koopmans MP, Osterhaus AD, Schurch AC (2014). Assembly of viral genomes from metagenomes. Front Microbiol.

[29] Sijmons S, Thys K, Corthout M, Van Damme E, Van Loock M, Bollen S, Baguet S, Aerssens J, Van Ranst M, Maes P (2014). A method enabling high-throughput sequencing of human cytomegalovirus complete genomes from clinical isolates. PLoS One.

[30] Kimura M (1980). A simple method for estimating evolutionary rates of base substitutions through comparative studies of nucleotide sequences. J Mol Evol.

[31] Donaldson EF, Harrington PR, O'Rear JJ, Naeger LK (2015). Clinical evidence and bioinformatics characterization of potential hepatitis C virus resistance pathways for sofosbuvir. Hepatology.

[32] Sims D, Sudbery I, Ilott NE, Heger A, Ponting CP (2014). Sequencing depth and coverage: key considerations in genomic analyses. Nat Rev Genet.

[33] Widasari DI, Yano Y, Heriyanto DS, Utsumi T, Yamani LN, Rinonce HT, Wasityastuti W, Lusida MI, Soetjipto OR (2014). A deep-sequencing method detects drug-resistant mutations in the hepatitis B virus in Indonesians. Intervirology.

[34] Bolcic F, Sede M, Moretti F, Westergaard G, Vazquez M, Laufer N, Quarleri J (2012). Analysis of the PKR-eIF2alpha phosphorylation homology domain (PePHD) of hepatitis C virus genotype 1 in HIV-coinfected patients by ultra-deep pyrosequencing and its relationship to responses to pegylated interferon-ribavirin treatment. Arch Virol.

[35] Dinis JM, Florek NW, Fatola OO, Moncla LH, Mutschler JP, Charlier OK, Meece JK, Belongia EA, Friedrich TC (2016). Deep sequencing reveals potential antigenic variants at low frequencies in influenza a virus-infected humans. J Virol.

[36] Wilker PR, Dinis JM, Starrett G, Imai M, Hatta M, Nelson CW, O'Connor DH, Hughes AL, Neumann G, Kawaoka Y (2013). Selection on haemagglutinin imposes a bottleneck during mammalian transmission of reassortant H5N1 influenza viruses. Nat Commun.

[37] Murphy E, Yu D, Grimwood J, Schmutz J, Dickson M, Jarvis MA, Hahn G, Nelson JA, Myers RM, Shenk TE (2003). Coding potential of laboratory and clinical strains of human cytomegalovirus. Proc Natl Acad Sci U S A.

[38] Murrell I, Wilkie GS, Davison AJ, Statkute E, Fielding CA, Tomasec P, Wilkinson GW, Stanton RJ (2016). Genetic stability of bacterial artificial chromosome-derived human cytomegalovirus during culture in vitro. J Virol.

[39] Smith IL, Taskintuna I, Rahhal FM, Powell HC, Ai E, Mueller AJ, Spector SA, Freeman WR (1998). Clinical failure of CMV retinitis with intravitreal cidofovir is associated with antiviral resistance. Arch Ophthalmol.

[40] Renzette N, Gibson L, Bhattacharjee B, Fisher D, Schleiss MR, Jensen JD, Kowalik TF (2013). Rapid intrahost evolution of human cytomegalovirus is shaped by demography and positive selection. PLoS Genet.

[41] Nei M, Li WH (1979). Mathematical model for studying genetic variation in terms of restriction endonucleases. Proc Natl Acad Sci U S A.

[42] Zhu T, Mo H, Wang N, Nam DS, Cao Y, Koup RA, Ho DD (1993). Genotypic and phenotypic characterization of HIV-1 patients with primary infection. Science.

[43] Vona B, Muller T, Nanda I, Neuner C, Hofrichter MA, Schroder J, Bartsch O, Lassig A, Keilmann A, Schraven S (2014). Targeted next-generation sequencing of deafness genes in hearing-impaired individuals uncovers informative mutations. Genet Med.

[44] Chan K, Roberts SA, Klimczak LJ, Sterling JF, Saini N, Malc EP, Kim J, Kwiatkowski DJ, Fargo DC, Mieczkowski PA (2015). An APOBEC3A hypermutation signature is distinguishable from the signature of background mutagenesis by APOBEC3B in human cancers. Nat Genet.

[45] Deuzing IP, Charpentier C, Wright DW, Matheron S, Paton J, Frentz D, van de Vijver DA, Coveney PV, Descamps D, ACH C (2015). Mutation V111I in HIV-2 reverse transcriptase increases the fitness of the nucleoside analogue-resistant K65R and Q151M viruses. J Virol.

[46] Howard JV (1998). The 2 × 2 table: a discussion from a bayesian viewpoint. Stat Sci.

[47] McDonlad J (2009). Handbook of Biological Statistics.

[48] McElroy K, Zagordi O, Bull R, Luciani F, Beerenwinkel N (2013). Accurate single nucleotide variant detection in viral populations by combining probabilistic clustering with a statistical test of strand bias. BMC Genomics.

[49] Singh T, Kurki MI, Curtis D, Purcell SM, Crooks L, McRae J, Suvisaari J, Chheda H, Blackwood D, Breen G (2016). Rare loss-of-function variants in SETD1A are associated with schizophrenia and developmental disorders. Nat Neurosci.

[50] Barbi M, Binda S, Caroppo S, Primache V, Dido P, Guidotti P, Corbetta C, Melotti D (2001). CMV gB genotypes and outcome of vertical transmission: study on dried blood spots of congenitally infected babies. J Clin Virol.

[51] Branas P, Blazquez-Gamero D, Galindo A, Prieto C, Olabarrieta I, Cuadrado I, Folgueira L (2015). Cytomegalovirus Genotype Distribution Among Congenitally and Postnatally Infected Patients: Association of Particular Glycoprotein (g) B and gN Types With Symptomatic Disease. Open Forum Infect Dis.

[52] Coaquette A, Bourgeios A, Dirand C, Varin A, Chen W, Herbein G (2004). Mixed cytomegalovirus glycoprotein B genotypes in immunocompromised patients. Clin Infect Dis.

[53] Nijman J, Mandemaker FS, Verboon-Maciolek MA, Aitken SC, van Loon AM, de Vries LS, Schuurman R (2014). Genotype distribution, viral load and clinical characteristics of infants with postnatal or congenital cytomegalovirus infection. PLoS One.

[54] Paradowska E, Jablonska A, Studzinska M, Suski P, Kasztelewicz B, Zawilinska B, Wisniewska-Ligier M, Dzierzanowska-Fangrat K, Wozniakowska-Gesicka T, Czech-Kowalska J (2013). Distribution of cytomegalovirus gN variants and associated clinical sequelae in infants. J Clin Virol.

[55] Pignatelli S, Lazzarotto T, Gatto MR, Dal Monte P, Landini MP, Faldella G, Lanari M (2010). Cytomegalovirus gN genotypes distribution among congenitally infected newborns and their relationship with symptoms at birth and sequelae. Clin Infect Dis.

[56] Ji YH, Sun ZR, Ruan Q, He R, Qi Y, Ma YP, Huang YJ (2006). High variability of human cytomegalovirus UL150 open reading frame in low-passaged clinical isolates. Chin Med Sci J.

[57] Jelcic I, Reichel J, Schlude C, Treutler E, Sinzger C, Steinle A (2011). The polymorphic HCMV glycoprotein UL20 is targeted for lysosomal degradation by multiple cytoplasmic dileucine motifs. Traffic.

[58] Britt WJ, Remington JS, Klein JO, Wilson CB, Nizet V, Maldonaldo Y (2011). Cytomegalovirus. Infectious Diseases of the Fetus and Newborn Infant.

[59] Arista S, De Grazia S, Giammanco GM, Di Carlo P, Iannitto E (2003). Human cytomegalovirus glycoprotein B genotypes in immunocompetent, immunocompromised, and congenitally infected Italian populations. Arch Virol.

[60] Burke HG, Heldwein EE (2015). Crystal structure of the human Cytomegalovirus glycoprotein B. PLoS Pathog.

[61] Speckner A, Glykofrydes D, Ohlin M, Mach M (1999). Antigenic domain 1 of human cytomegalovirus glycoprotein B induces a multitude of different antibodies which, when combined, results in incomplete virus neutralization. J Gen Virol.

[62] Spindler N, Diestel U, Stump JD, Wiegers AK, Winkler TH, Sticht H, Mach M, Muller YA (2014). Structural basis for the recognition of human cytomegalovirus glycoprotein B by a neutralizing human antibody. PLoS Pathog.

[63] Wiegers AK, Sticht H, Winkler TH, Britt WJ, Mach M (2015). Identification of a neutralizing epitope within antigenic domain 5 of glycoprotein B of human cytomegalovirus. J Virol.

[64] Frange P, Boutolleau D, Leruez-Ville M, Touzot F, Cros G, Heritier S, Moshous D, Neven B, Fischer A, Blanche S (2013). Temporal and spatial compartmentalization of drug-resistant cytomegalovirus (CMV) in a child with CMV meningoencephalitis: implications for sampling in molecular diagnosis. J Clin Microbiol.

[65] Meyer-Konig U, Vogelberg C, Bongarts A, Kampa D, Delbruck R, Wolff-Vorbeck G, Kirste G, Haberland M, Hufert FT, von Laer D (1998). Glycoprotein B genotype correlates with cell tropism in vivo of human cytomegalovirus infection. J Med Virol.

[66] Bootz A, Karbach A, Spindler J, Kropff B, Reuter N, Sticht H, Winkler TH, Britt WJ, Mach M (2017). Protective capacity of neutralizing and non-neutralizing antibodies against glycoprotein B of cytomegalovirus. PLoS Pathog.

[67] Marou E, Liaskos C, Simopoulou T, Efthymiou G, Dardiotis E, Katsiari C, Scheper T, Meyer W, Hadjigeorgiou G, Bogdanos DP (2017). Human cytomegalovirus (HCMV) UL44 and UL57 specific antibody responses in anti-HCMV-positive patients with systemic sclerosis. Clin Rheumatol.

[68] Perez-Carmona N, Martinez-Vicente P, Farre D, Gabaev I, Messerle M, Engel P, Angulo A. A Prominent Role of the Human Cytomegalovirus UL8 Glycoprotein in Restraining Proinflammatory Cytokine Production by Myeloid Cells at Late Times during Infection. J Virol. 2018;92(9):30. 10.1128/JVI.02229-17 PMC5899185 29467314

[69] Tadagaki K, Tudor D, Gbahou F, Tschische P, Waldhoer M, Bomsel M, Jockers R, Kamal M (2012). Human cytomegalovirus-encoded UL33 and UL78 heteromerize with host CCR5 and CXCR4 impairing their HIV coreceptor activity. Blood.

[70] Pokalyuk C, Renzette N, Irwin KK, Pfeifer SP, Gibson L, Britt WJ, Yamamoto AY, Mussi-Pinhata MM, Kowalik TF, Jensen JD (2017). Characterizing human cytomegalovirus reinfection in congenitally infected infants: an evolutionary perspective. Mol Ecol.

