# Correction to: Association of CMV genomic mutations with symptomatic infection and hearing loss in congenital CMV infection

**DOI:** 10.1186/s12879-020-4766-9

**Published:** 2020-02-10

**Authors:** G. Clement Dobbins, Amit Patki, Dongquan Chen, Hemant K. Tiwari, Curtis Hendrickson, William J. Britt, Karen Fowler, Jake Y. Chen, Suresh B. Boppana, Shannon A. Ross

**Affiliations:** 1 Department of Pediatrics, The University of Alabama School of Medicine, CHB 116, 1600 6th Avenue South, Birmingham, AL USA; 2 Department of Biostatistics, The University of Alabama School of Public Health, Birmingham, AL USA; 3 Informatics Institute, The University of Alabama at Birmingham, Birmingham, AL USA; 4 Department of Medicine, The University of Alabama at Birmingham, Birmingham, AL USA; 5 Department of Microbiology, The University of Alabama at Birmingham, Birmingham, AL USA

**Correction to: BMC Infect Dis**

**https://doi.org/10.1186/s12879-019-4681-0**

After publication of the original article [1], we were notified that Fig. 3 has “Fig. 1” posted on the top of it and Figs. 4 and 5 have “Genomic Position” in a different spot.

Below you can find the correct figures:

**Fig. 3:**
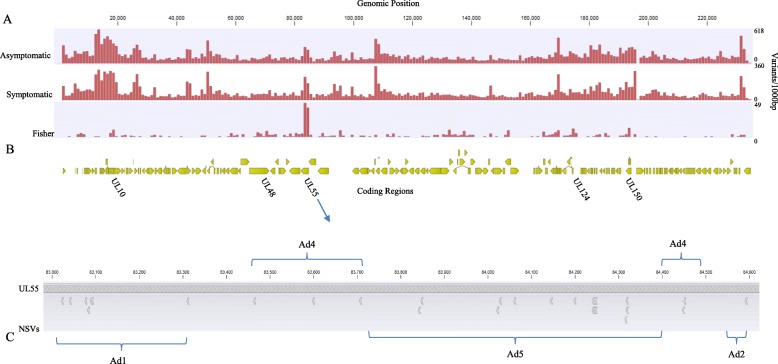
CMV variants in symptomatic and asymptomatic children. **a** Variant density of CMV genomes isolated from newborns (17 asymptomatic, 13 symptomatic) calculated in 1000 bp windows with Merlin as the reference strain. **b** Variants more frequent in symptomatic infection (Fisher’s exact test *p* < 0.05) are plotted with the genome position. The coding regions with the highest number of such variants are listed in their relative genomic position. **c** UL55 NSVs in relation to known antigenic domains (AD). Top panel shows the amino acid sequence of CMV strain Merlin. Bottom panel shows where NSVs (indicated by arrows below the reference strain) are more likely seen in infants with symptomatic congenital CMV in relation to antigenic domains

**Fig. 4:**
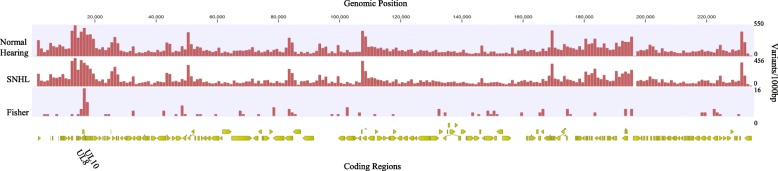
CMV variation from children with normal hearing and SNHL. Variant density of CMV genomes isolated from 17 children with normal hearing and 13 children with SNHL calculated in a 1000 bp sliding window with Merlin as the reference strain. Variants more frequent in viruses from children with SNHL (Fisher’s exact test *p* < 0.05) are plotted with the genome position. The coding regions with the highest number of variants are listed with the genomic position

**Fig. 5:**
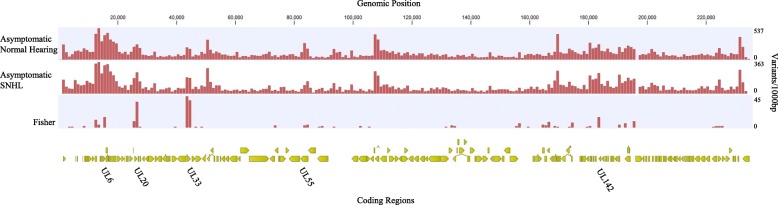
CMV variants from asymptomatic children with normal hearing and SNHL. Variant density of CMV genomes isolated from 11 asymptomatic newborns with normal hearing and 6 asymptomatic newborns that developed SNHL calculated in a 1000 bp sliding window with Merlin as the reference strain. Regions with variants more frequent in viruses from children with asymptomatic infection and SNHL (Fisher’s exact test *p* < 0.05) are plotted with the genome position

The original article has been corrected.
