# Supplementary material for: Association of CMV genomic mutations with symptomatic infection and hearing loss in congenital CMV infection
Source: BMC Infect Dis. 2019 Dec 10;19:1046. doi: 10.1186/s12879-019-4681-0 (PMC6905059; doi:10.1186/s12879-019-4681-0)
Supplement: Supplementary file 1 — Additional file 1: Figure S1. All unique CMV variants in the 30 newborns (top panel) along with those divided into each group. The number of unique variants are listed in each group on the left with the maximum number of variants in any 1000 base pair window on the right. Bottom panel is schematic representation of CMV coding regions based on the reference strain Merlin along with regions with the most variation. [file 12879_2019_4681_MOESM1_ESM.pptx]

## Slide 1
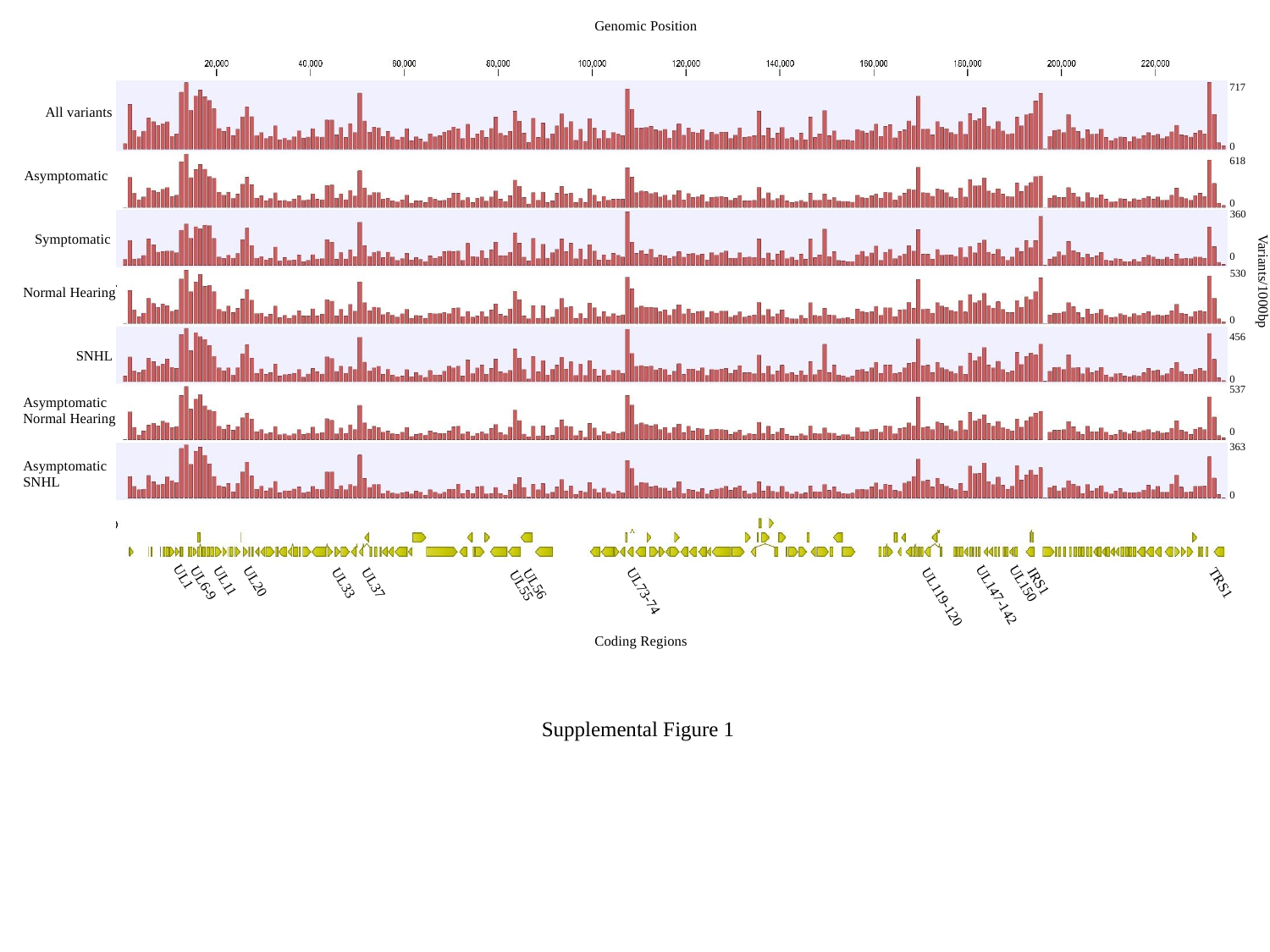

Genomic Position
717
All variants
0
618
Asymptomatic
0
360
Symptomatic
0
530
Normal Hearing
Variants/1000bp
0
456
SNHL
0
537
Asymptomatic Normal Hearing
0
363
Asymptomatic SNHL
0
UL1
UL6-9
UL11
UL20
UL33
UL37
UL73-74
UL56
UL55
UL147-142
UL150
UL119-120
IRS1
TRS1
Coding Regions
Supplemental Figure 1
